# Competitive Degradation of Steroid Estrogens by Potassium Permanganate Combined with Ultrasound

**DOI:** 10.3390/ijerph121214995

**Published:** 2015-12-04

**Authors:** Jing Deng, Kai Tang, Shijun Zhu, Xiaoyan Ma, Kejia Zhang, Yali Song, Xueyan Li, Qingsong Li, Zhenhua Liu, Kejin Zhou

**Affiliations:** 1 College of Civil Engineering and Architecture, Zhejiang University of Technology, Hangzhou 310014, China; seudjing@163.com (J.D.); kaitang57@gmail.com (K.T.); 2 School of Municipal and Environmental Engineering, Harbin Institute of Technology, Harbin 150001, China; 3 Department of Civil Engineering, Zhejiang University, Hangzhou 310058, China; zkj1025@163.com (K.Z.); 4 School of Civil Engineering and Architecture, Zhejiang University of Science and Technology, Hangzhou 310023, China; yali_song@sina.com; 5 School of Environmental Science and Engineering, Suzhou University of Science and Technology, Suzhou 215009, China; lxyhit@sina.com; 6 Water Resources and Environmental Institute, Xiamen University of Technology, Xiamen 361005, China; leetsingsong@sina.com; 7 Department of Municipal Engineering, Zhejiang University of Water Resource and Electric Power, Hangzhou 361018, China; liuzhh@zjwchc.com; 8 Zhejiang Province Environmental Monitoring Center, Hangzhou 310012, China; zhoukj@163.com

**Keywords:** coexisting pollutant, competitive degradation, drinking water treatment, KMnO_4_/ultrasound, steroid estrogens

## Abstract

The occurrence of natural estrogens including estrone (E1), 17β-estradiol (E2), and synthetic 17α-ethinylestradiol (EE2), which can be excreted by both humans and animals, and can enter the aqueous environment along with the discharge of domestic sewage, is a major concern since this may represent a serious health risk to humans even at extremely trace levels (ng·L^−1^). Simultaneous degradation of three coexisting steroid estrogens (SEs) in aqueous solutions by coupled ultrasound and KMnO_4_ systems (KMnO_4_/ultrasound) were investigated to find out whether there is a competitive degradation of multiple contaminants or not. Results indicate that the degradation ratios of target SEs were all more than 50% after 120 min reaction contact, greatly enhanced when compared with the single KMnO_4_ (2 mg·L^−1^) oxidation of E2 (37.0%), EE2 (34.4%), and E1 (34.0%), and the single sonochemical oxidation of E2 (37.1%), EE2 (31.1%), and E1 (29.7%). In the adopted processes, the degradations of SEs fit the first-order kinetic reaction, with different reaction rates. Kinetic parameters revealed there was little difference between coexisting SEs, which means there was almost no competitive degradation. The removal efficiency and degradation rate of SEs in natural water was higher than those in pure water, which suggested that the coupled KMnO_4_/ultrasound technology had prospective applications in the removal of complex contaminants in actual drinking water treatment.

## 1. Introduction

Steroid estrogens (SEs) are kinds of micropollutants detected in aqueous circumstances that are attracting wide concern since they have ten thousand to ten million times the estrogenic activity of other endocrine disrupting chemicals (EDCs) [1]. SEs, characterized with polytype, prevalent distribution, low concentrations, great risk, and difficult degradation, may inhibit or undermine the normal function of endocrine and other vital systems through interfering with synthesis, secretion, transport, binding, action or elimination of natural hormones in the body which are responsible for the maintenance of homeostasis, adjustment, reproduction, development, and/or behavior [2,3,4,5,6,7,8]. Steroid estrogen (SEs) including natural estrogenic steroids such as estrone (E1), 17β-estradiol (E2), estriol (E3), and synthetic steroid 17α-ethynylestradiol (EE2) in aqueous environments are organics of tetracyclic molecules which have a benzene ring and conjugated double bond with a molecular weight between 200 and 400, normally [9].

Natural steroid estrogens can enter the aqueous environment through the discharge of the wastewater-containing metabolism of humans and livestock herding, such as urine and excrement. Synthetic steroids usually originate from medication (e.g., oral contraceptive) produced in pharmaceutical enterprises with industrial effluents emission. Recently, SEs have been detected in sewage, surface water, underground water, and even sources of drinking water [10,11,12]. An extensive survey of steroid estrogens was conducted for sources water of Hangzhou City, China, and the results showed traces of EE2 (1.17–3.35 ng·L^−1)^, and E2 (0.132 ng·L^−1^) existing in Qiantang River [10]. In the north of Germany, estrogens were detected in the range of 0.2–0.6 ng·L^−1^ for E1 and 0.2–2.1 ng·L^−1^ for E2. E1, E2 and EE2 were found in a reservoir of source water in Shanghai with a concentration range of 1.0–110.0, 0.0–90.0, and 0.8–80.0 ng·L^−1^ respectively [12]. Those SE compounds in source water may penetrate conventional treatment units (e.g., coagulation/sedimentation, filtration, and chlorination), and then finally get to the faucet of users, bringing with them potential negative effects. Therefore, it is important to find the proper methods to reduce the contamination arousing public concern.

Activated carbon (AC) as an effective adsorbent is obviously efficient for the removal of SEs at high concentrations [13,14,15,16,17]. However, when source water is contaminated by complex contaminants besides SEs, the adsorption efficiency to extracted SEs will be influenced by the matrix, such as NOM or other micropollutants, so longer contact times and a large quantity of AC should be needed to get favorable adsorption [17]. Biological degradation and enzymatic reactions were more often employed in the removal of SEs that were not suitable for drinking water treatment, usually in the sewage treatment process due to the slow reduction rates, long treatment time, and incomplete decomposition [18]. Recently, there have been numerous studies on the removal of estrogens through chemical oxidation [19], advanced oxidation [20,21,22], and photocatalysis [23] in combination with UV and strong oxidizers [24], but the most focus is on the analysis of the degradation rate, efficiencies, and influential factors for a single target pollutant while ignoring simultaneous degradation of pollutants in multiple contamination matrices. The objective here is to summarize the simultaneous or probable competitive elimination of coexisting SEs in the KMnO_4_/ultrasound treatment process by using simplified models.

## 2. Materials and Methods

### 2.1. Chemicals

Standard estrone (E1), 17β-estradiol (E2), and 17α-ethinyl estradiol (EE2) were purchased from Sigma-Aldrich. Methanol (HPLC grade) was obtained from Tedia, and acetonitrile (HPLC grade) was supplied by Merck. Pure water was obtained through the Milli-pore equipment. The KMnO_4_ and sodium thiosulfate were commercially available analytical-grade products.

### 2.2. Ultrasonic Apparatus and Reactor Facility

The ultrasonic generator FS-300 (Sonxi, Shanghai, China) can operate either continuously or in a pulsed mode at a fixed frequency of 20 kHz and a variable power output of up to 300 W. Ultrasound irradiation was emitted through a tip 1 cm in diameter. The tip was fixed on a tripod, which was installed centrally in a 10 L cylindrical reactor to make sure that the tip immersed 5 cm away from the bottom.

The reactor was operated in a recirculation mode at a flow velocity of 2 L·min^−1^ fixed by a pump installed outside of the reactor, which plays a role of blending and stirring. Plastic tubes were fixed around the external part of the reactor, and tap water was kept running through during experiments to maintain a constant temperature.

### 2.3. Analytical Methods

**Figure 1:**
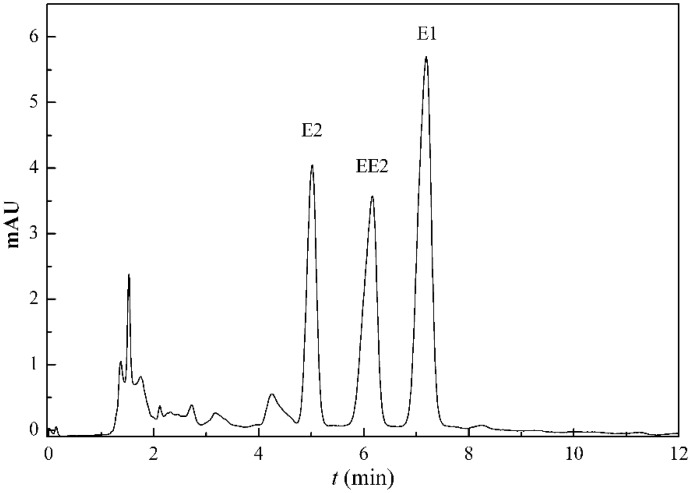
Liquid chromatography of three kinds of steroid estrogens.

A 12 solid phase extraction device (CNW, USA) with C18 solid phase extraction columns (CNW, USA) was used to extract SEs from aqueous solutions. Agilent1200 HPLC with an ultraviolet detector set at 220 nm was used to determine the concentrations of SEs in samples with an Eclipse XDB-C18 column (5 μm, 46 × 150 mm, USA). Sample injections were achieved by an SIJ-20A auto injection system. The mobile phase water/acetonitrile (50/50, v/v) was run in an isocratic mode at a constant flow rate of 0.8 mL·min^−1^, and the column was maintained at a constant of 30 °C. The injection volume was 20 μL. Liquid chromatography of three kinds of steroid estrogens is shown in Figure 1. The peaks of SEs were shown in a sequence of E2, EE2, and E1, and the retention times were 5.0, 6.1, and 7.2 min, respectively.

### 2.4. Experimental Procedure

Solutions of SEs were prepared by deionized water in the reactor, adjusted to optimum pH 7.6 with 0.1 mol·L^−1^ HCl and 0.1 mol·L^−1^ NaOH, according to the previous description of Ma *et al.* [25]. Samples were collected to determine the initial concentration (C_0_) of SEs after sufficient mixture. Except those which took temperature variation into consideration, the experiments were performed at room temperature (23 ± 2 °C). Once the oxidation process was started; sequence samples (100 mL) were collected into beakers (150 mL) at 2, 5, 10, 15, 30, 50, 80, and 120 min intervals with substantial sodium thiosulphate immediately added to quench the reaction. Residual concentrations were analyzed as soon as possible after pretreatment, and were otherwise stored at −4 °C in darkness.

Water samples (100 mL) were first concentrated by solid phase extraction (SPE) using C18 cartridges (3 mL, 500 mg), which were pre-conditioned with 10 mL of methanol, 10 mL of acetonitrile, and 20 mL of pure water in sequence at a flow of 3 mL·min^−1^. After samples had been loaded entirely, the cartridges were then eluted with 3 mL of acetonitrile. The eluents were dried under a gentle nitrogen purge, then redissolved in 1 mL acetonitrile, and finally were analyzed by HPLC. The residual water samples were stored at −4 °C in darkness to be measured for other water quality parameters. The single ultrasound process was applied at 210 W power and 20 kHz frequency. The same parameters were adopted in combined KMnO_4_/ultrasound process and the KMnO_4_ dosage was designed to be 2mg·L^−1.^

## 3. Results and Discussion

### 3.1. Simultaneous Degradation of SEs by Potassium Permanganate Oxidation

**Figure 2:**
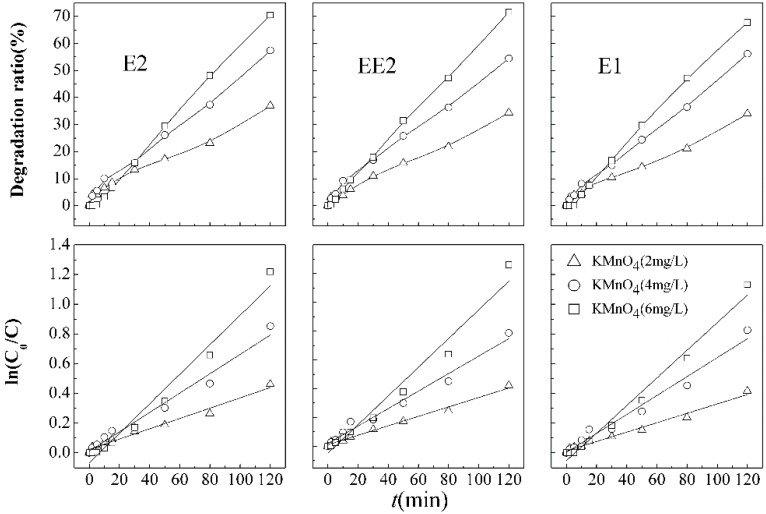
Degradation curves and first order kinetic models of SEs under different dosage of potassium permanganate.

Figure 2 shows the simultaneous degradation kinetics models of mixed E1, E2, and EE2, with initial concentrations of 13, 17, 25 μg·L^−1^ respectively, in single potassium permanganate oxidation processes with dosages of 2, 4, and 6 mg·L^−1^.

In coexisting competitive degradation systems, E1, E2, and EE2 respectively had low removal rates of 37.0%, 34.4%, and 34.0% after 120 min of contact time with a minimum KMnO_4_ dosage of 2 mg·L^−1^. The results suggested that E2 could be slightly better degraded than the other two SEs at the degradation level. When the amount of oxidant increased to 4 mg·L^−1^, the removal efficiencies of SEs obviously enhanced, to approximately 57.4% (E2), 54.5% (EE2), and 56.2% (E1). With KMnO_4_ dosages continually increasing to 6 mg·L^−1^, the removal efficiencies increased up to 71.6% (E2), 70.5% (EE2), and 67.8% (E1). The results revealed a positive relationship between removal efficiencies of SEs and KMnO_4_ dosage. The contaminants removal mechanisms by KMnO_4_ include conventional oxidation and catalysis/adsorption generated by the reaction products of Mn^2+^ and MnO_2_ [26]. Numerous studies demonstrated that pH had a significant effect on the redox potential of permanganate with 1.51 V, 0.588 V, and 0.564 V for acidic, neutral, and alkaline conditions, respectively [27,28]. The following Equations (1)–(3) apply:

(1) $${\text{MnO}}_{\text{4}}^{\text{-1}}{\text{ + 8H}}^{+}{\text{ + 5e}}^{\text{-}}{\text{ = Mn}}^{\text{2}+}{\text{ + 4H}}_{\text{2}}\text{O}$$

(2) $${\text{MnO}}_{\text{4}}^{\text{-1}}{\text{ + 2H}}_{\text{2}}{\text{O + 3e}}^{\text{-}}{\text{ = MnO}}_{\text{2}}{\text{ + 4OH}}^{\text{-}}$$

(3) $${\text{2MnO}}_{\text{4}}^{\text{-1}}{\text{ + 3Mn}}^{\text{2}+}{\text{ + 2H}}_{\text{2}}{\text{O = 5MnO}}_{\text{2}}{\text{ + 4H}}^{+}$$

MnO_2_ as a catalyst produced during reaction improved the oxidation rate, and the colloids compounded by MnO_2_ had a larger surface area to adsorb organisms in aqueous systems [22,29].

Under simulated combination pollutant conditions, the degradation of SEs follows pseudo-first-order reaction kinetics under various KMnO_4_ doses, and parameters are shown in Table 1. In the KMnO_4_ oxidation process, considering the removal efficiency, reaction rate, and half-life, superior degradation of E2 was recognized and supposed to be attributed to the lower initial concentration.

Since KMnO_4_ can induce color into water, the lowest KMnO_4_ dosage (2 mg·L^−1^) was selected in the subsequent experiments for further understanding of the simultaneous estrogen removal in the KMnO_4_/ultrasound system.

**Table 1:** Parameters of the kinetic model of degradation of SEs under single potassium permanganate treatment with different dosages.

Steroid Estrogens	KMnO_4_/(mg·L^−1^)	Kinetic Equation	Reaction Rate Constant K/min^−1^	R^2^	Half-Life *t*_1/2_/min
E2	2	ln(C_0_/*C*) = 0.0035*t* + 0.025	0.0035	0.978	193
	4	ln(C_0_/*C*) = 0.0065*t* + 0.013	0.0065	0.975	105
	6	ln(C_0_/*C*) = 0.0099*t* − 0.065	0.0099	0.974	76
EE2	2	ln(C_0_/*C*) = 0.0033*t* + 0.011	0.0033	0.988	208
	4	ln(C_0_/*C*) = 0.0061*t* + 0.090	0.0061	0.979	99
	6	ln(C_0_/*C*) = 0.0100*t* − 0.049	0.0100	0.970	74
E1	2	ln(C_0_/*C*) = 0.0032*t* + 0.015	0.0032	0.980	215
	4	ln(C_0_/*C*) = 0.0064*t* + 0.049	0.0064	0.973	108
	6	ln(C_0_/*C*) = 0.0093*t* − 0.052	0.0093	0.984	80

### 3.2. Simultaneous Degradation of SEs by Ultrasound

Numerous researchers indicated that the organic matters could be removed by single ultrasound to various degrees. Influencing factors were reported to have a strong effect on degradation of organic contaminants [30,31,32,33,34].

**Figure 3:**
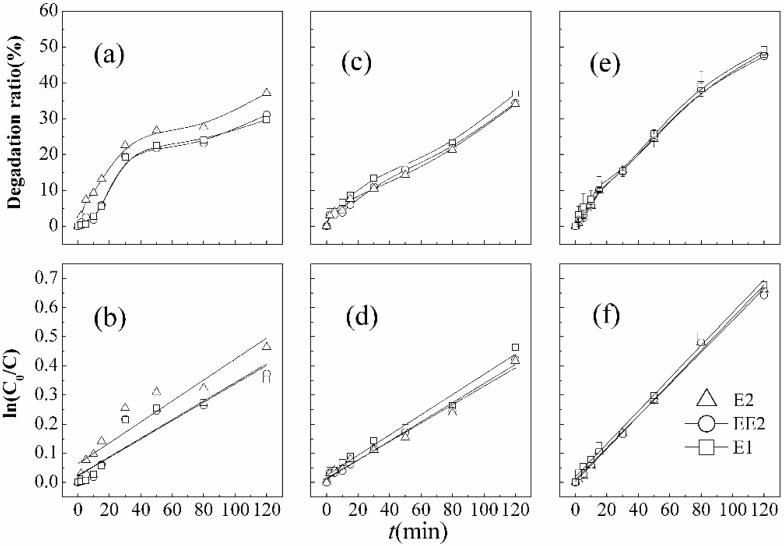
Degradation curves and first order kinetic models of SEs in different treatment processes ((**a**), (**b**) single ultrasound; (**c**), (**d**) single KMnO_4_; (**e**), (**f**) combined KMnO_4_/ultrasound).

Removal efficiencies of 37.1% (E1), 31.1% (E2), and 29.7% (EE2) were obtained by using single ultrasound irradiation as shown in Figure 3. Results were obtained after 120 min of contact, and the degradation of SEs fitted pseudo-first-order reaction kinetics. Sonochemical reaction involved the complex phenomenon of oxidation derived from cavitations. Cavitation bubbles grow from existing gas nuclei, oscillate, and collapse to generate high temperatures and pressures that induce target compound pyrolysis inside the bubble/or at the bubble–liquid interface at exceedingly short times [25,35]. Mechanisms of ultrasonic degradation of organic contaminants had been proposed as pyrolytic decomposition, hydroxyl radical oxidation, plasma chemistry, and supercritical water oxidation [35]. When the sound waves pass through the liquid, they create cavities due to oscillating acoustic pressures. The dissolved gases, organic compounds, and water vapor can diffuse into the cavities from bulk solutions. These cavities grow in size and ultimately implode, generating temperatures as high as 5200 K and pressures higher than 1000 atm inside the collapsing cavity, and about 1900 K in the interfacial region between the solution and the collapsing bubble. The destruction of organic pollutants occurs via several mechanisms. The organic pollutant inside the cavity and in the interfacial region (cavity–liquid) can undergo thermal degradation (pyrolysis or combustion reactions if oxygen is present during the implosion). Another mechanism is that free radicals (•OH, •H, •HO_2_) formed due to thermolysis of the water molecules can react with the organics in the interfacial region or in the bulk solution near the interface [36]. Free radicals can subsequently react with steroid estrogens in the aqueous phase, hydroxyl radicals (•OH) as the representative, are strong oxidizers with a standard redox potential (*E*_0_) of 2.80 eV. As the circumstance of temperature and pH is fixed, the organic contaminants are removed mainly depending on the acoustic cavitation pyrolysis due to a few free radicals produced by single ultrasound in the pure water model. Furthermore, acoustic cavitations were generated on a tiny scale of ultrasonic source, and the energy utilization ration is merely 15% [37].

It can be recognized from Figure 3a,d that E2 takes advantage with a higher degradation ratio constant over EE2 and E1, which is more apparent in the sonic degradation process than in KMnO_4_ oxidation. Considering the mechanism of ultrasound irradiation, hydrophobicity might play an important role in the degradation, besides initial concentration. Hydrophobicity has a positive effect on the pyrolysis reaction [36]. Due to their hydrophobicity, the estrogens would tend to diffuse into the cavity-liquid interface. The supercritical environment produced in the interfacial region would increase the solubility of estrogens. Three target estrogens are hydrophobic compounds, with low octanol-water partition coefficients (logK_ow_). The hydrophobicity of E2 is slightly stronger than EE2 and E1, and thus it is easier to enter into the cavitation bubbles and be decomposed in the coexisting system.

The emission frequency of the ultrasonic generator used in this experiment was 20 kHz, which was much lower than those reported by other researchers (ranging from 200 to 1200 kHz). The increase of frequencies could definitely improve the degradation ratio of organic contaminants because of more energy consumption [38,39,40]. Above results showed that single ultrasound or KMnO_4_ were inefficient in SEs degradation, while combined KMnO_4_/ultrasound could improve the degradation of SEs significantly [25,41].

### 3.3. Simultaneous Degradation of SEs by KMnO_4_/Ultrasound

#### 3.3.1. Degradation Efficiency of SEs in Different Treatment Process

Degradation of SEs fit pseudo-first-order kinetics in each treatment process. The removal curves for the different treatment processes are shown in Figure 3. After a 120 min reaction, the degradation efficiencies of E2 (49.2%), EE2 (48.4%), and E1 (47.4%) by ultrasound/KMnO_4_ were higher than those by single treatment processes, which reveal more effectiveness of combined ultrasound/KMnO_4_ process.

(4) $$-\frac{dC}{dt}=kC\Leftrightarrow \text{ln}\frac{C_{0}}{C}=kt$$

where *k* is an apparent rate constant, incorporating the constant concentration of oxidizing agents. The rate constants of oxidation (*k*_KMnO_4__), ultrasonic irradiation(*k*_US_), and their combination (*k*_US/KMnO_4__) can be calculated according to Equation (4). The degree of synergy S can then be assessed as follows [30]:

(5) $$\%\text{S}=\frac{k_{\text{US/KMn}O_{4}}-(k_{\text{US/KMn}O_{4}}+k_{\text{US}})}{k_{\text{US/KMn}O_{4}}}\times 100$$

In this case, S < 0 indicates that there is not a good synergy between them, possibly due to the low frequencies of the ultrasonic generator in the experiments. It is generally known that radical generation by ultrasound is more effective at higher ultrasound frequencies. At lower frequencies, the cavitation effect is the dominant factor. Frontistis *et al.* investigated the photocatalytic (UV-A/TiO_2_) degradation of 17-ethynylestradiol (EE2) in environmental matrices, and showed that there is a two-way synergy between them (S = 44.3%), while the horn-type sonicator operating at 80 kHz was employed for sonophotocatalytic experiments [42].

Reaction rate constants in Table 2 manifest that there are minor differences between the degradation rate of E1, E2, and slightly lower of EE2. Accordingly, the results elucidate that there is almost no obvious competitive degradation of target SEs under the condition of lower initial concentrations.

The mechanisms of the synergetic effect of ultrasounds and KMnO_4_ could be explained as follows Ma *et al.* [41].

H_2_O produces H_2_O_2_, which undergoes partial dissociation in the presence of ultrasound.

(6) $${\text{H}}_{2}{\text{O}}_{2}+{\text{H}}_{2}\text{O}―\text{H}{\text{O}}_{\text{2}}^{-}+{\text{O}}_{\text{3}}^{+}$$

The H_3_O^+^ makes the solution acidic, which improves the generation of •OH decomposed by KMnO_4_.

(7) $$\text{4}{\text{MnO}}_{\text{4}}^{\text{-1}}{\text{ + 4H}}^{+}―{\text{4MnO}}_{\text{2}}↑+{\text{2H}}_{\text{2}}\text{O}+{\text{3O}}_{\text{2}}$$

(8) $${\text{O}}_{\text{2}}+)))—•\text{O}+•\text{O}$$

(9) $$\text{O}•+{\text{H}}_{\text{2}}\text{O}—•\text{OH}+•\text{OH}$$

Furthermore, the redox reaction occurs between H_2_O_2_ and KMnO_4_. Mn^2+^ and MnO_2_, produced in the reaction, have a strong catalytic effect on cavitations, thus promoting further degradation of the organic contaminants. Accordingly, KMnO_4_ can improve the ultrasound to produce •OH in an aqueous environment.

(10) $$\text{2}{\text{MnO}}_{\text{4}}^{\text{-1}}+\text{5}{\text{H}}_{\text{2}}{\text{O}}_{\text{2}}+{\text{6H}}^{+}―\text{2}{\text{Mn}}^{+}+\text{5}{\text{O}}_{\text{2}}+\text{8}{\text{H}}_{\text{2}}\text{O}$$

(11) $$\text{2}{\text{MnO}}_{\text{4}}^{\text{-1}}+\text{3}{\text{H}}_{\text{2}}{\text{O}}_{\text{2}}+―\text{2}\text{Mn}{\text{O}}_{\text{2}}+\text{3}{\text{O}}_{\text{2}}+\text{2}{\text{OH}}^{-}+\text{2}{\text{H}}_{\text{2}}\text{O}$$

Permanganate was usually applied as a pre-oxidant in the drinking water treatment processes. The MnO_2_ as a type of light yellow granule, can be removed easily in the following traditional treatment units, e.g., coagulation/sedimentation, filtration, *etc.* Considering the residual manganese and color problem, dosages of permanganate were always controlled to under a certain value.

**Table 2:** Degradation kinetic parameters of SEs under different processes.

Steroid Estrogens	Treatment Technologies	Kinetic Equation	Reaction Rate Constant K/min^−1^	R^2^	Half-Life *t*_1/2_/min
E2	KMnO_4_ (2mg·L^−1^)	ln(C_0_/*C*) = 0.0035*t* + 0.025	0.0035	0.978	191
	Pure ultrasound	ln(C_0_/*C*) = 0.0036*t* + 0.064	0.0036	0.888	175
	KMnO_4_/ultrasound	ln(C_0_/*C*) = 0.0056*t* + 0.003	0.0056	0.996	123
EE2	KMnO_4_ (2mg·L^−1^)	ln(C_0_/*C*) = 0.0033*t* + 0.011	0.0033	0.988	207
	Pure ultrasound	ln(C_0_/*C*) = 0.0032*t* + 0.024	0.0032	0.874	210
	KMnO_4_/ultrasound	ln(C_0_/C) = 0.0055*t* + 0.010	0.0055	0.994	125
E1	KMnO_4_ (2mg·L^−1^)	ln(C_0_/*C*) = 0.0032*t* + 0.015	0.0032	0.980	212
	Pure ultrasound	ln(C_0_/*C*) = 0.0032*t* + 0.023	0.0032	0.852	210
	KMnO_4_/ultrasound	ln(C_0_/*C*) = 0.0056*t* + 0.020	0.0056	0.993	120

#### 3.3.2. Effects of Initial Concentration of SEs

Sample solutions of mixed SEs were prepared in concentrations of approximately 50, 100, and 500 μg·L^−1^. The impact of the initial concentration on the SEs’ simultaneous degradation in a KMnO_4_/ultrasound process were investigated, with KMnO_4_ (2 mg·L^−1^), ultrasonic power (210 W) and a frequency of (20 kHz). Degradation kinetic models of SEs under different initial concentrations were showed in Table 3.

**Table 3:** Parameters of the degradation kinetic model of SEs under different initial concentrations.

Steroid Estrogens	Initial Concentration (μg·L^−1^)	Kinetic Equation	Reaction Rate Constant K/min^−1^	R^2^	Half-Lif *t*_1/2_/min
E2	50	ln(C_0_/*C*) = 0.0056*t* + 0.003	0.0056	0.996	123
	100	ln(C_0_/*C*) = 0.0041*t* + 0.020	0.0041	0.984	163
	500	ln(C_0_/*C*) = 0.0029*t* + 0.019	0.0029	0.972	230
EE2	50	ln(C_0_/*C*) = 0.0055*t* + 0.010	0.0055	0.994	125
	100	ln(C_0_/*C*) = 0.0039*t* + 0.032	0.0039	0.964	169
	500	ln(C_0_/*C*) = 0.0024*t* + 0.022	0.0024	0.944	212
E1	50	ln(C_0_/*C*) = 0.0056*t* + 0.020	0.0056	0.993	120
	100	ln(C_0_/*C*) = 0.0038*t* + 0.042	0.0038	0.959	172
	500	ln(C_0_/*C*) = 0.0024*t* + 0.025	0.0024	0.965	282

**Figure 4:**
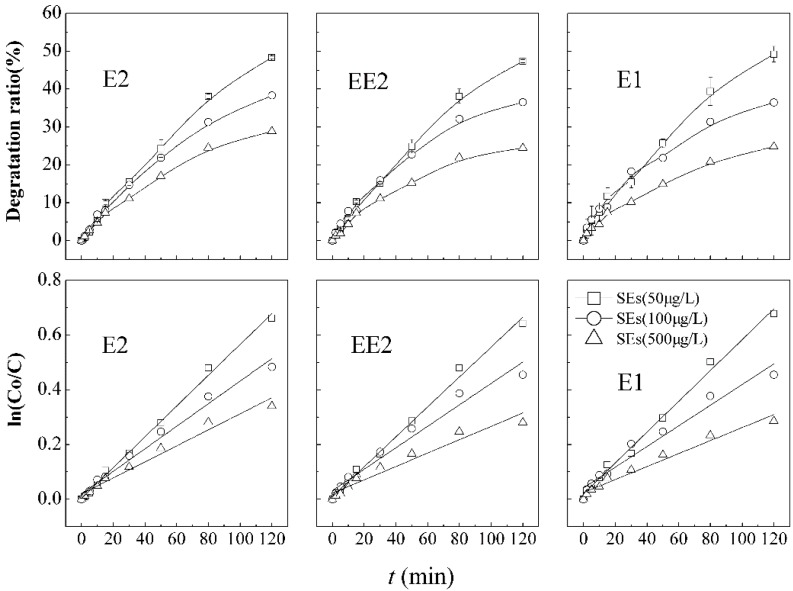
Degradation curves and first order kinetic models of SEs in combined processes under different initial concentrations.

As shown in Figure 4, when the initial concentration of SEs is 100 μg·L^−1^, the degradation efficiencies of E2, EE2, and E1 are 38.3%, 36.6%, and 36.5%, respectively, with little difference in the coexisting system after 120-min-contact. However, it was observed that E2, EE2, and E1 were only removed by 28.8%, 24.4%, and 24.8%, respectively, when the initial concentration of SEs increases to about 500 μg·L^−1^ with the same reaction time. Simultaneous degradation of the coexisting combinations of estrogens in KMnO_4_/ultrasound system follows the apparent first-order kinetics shown in Figure 4. The degradation ratio and removal rate decrease with increasing initial concentrations of estrogens, which is similar to the conclusions of He *et al.* who used combined Fe(III)/H_2_O_2_ technology [43]. Low concentration favors the degradation of SEs. The reason may be due to the low concentration accompanied by preferable contact with free hydroxyl radicals (•OH) and little oxidant demand.

#### 3.3.3. Analysis of Competitive Degradation of Coexisting Steroid Estrogens

**Figure 5:**
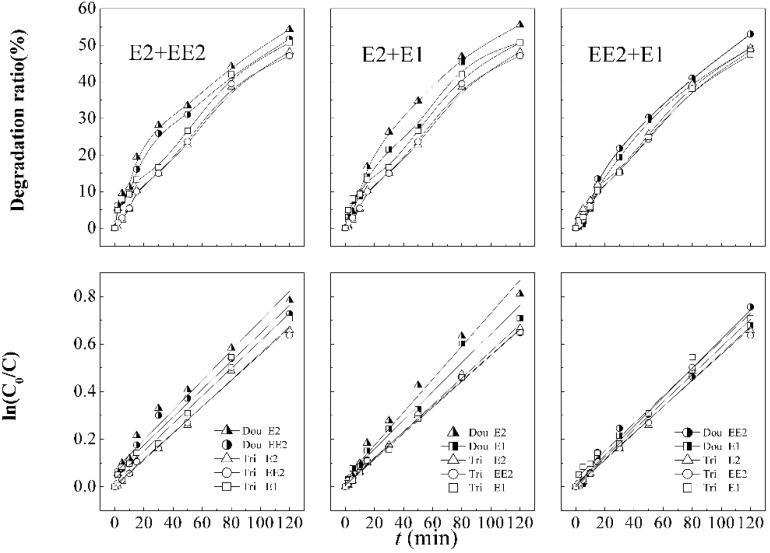
Degradation curves and first order kinetic models of dual- and tri-mixed SEs in the combination process.

The simultaneous and competitive degradation of SEs in the ultrasound/KMnO_4_ process was further investigated by serial pair combinations of E2, EE2, and E1 and results were showed in Figure 5 and Table 4. Some researches pointed out that an estrogenic coexisting system had more adverse influences on the ecological environment [7]. Additionally, it is supposed that coexisting SEs have mutual effects on each other in the decomposition process. Therefore, different coexisting combinations of estrogens (E2 and EE2; E2 and E1; EE2 and E1) were designed to summarize simultaneous or probable rules of competitive elimination and the impacts on each other. The concentration and percentage of each constituent were nearly identical to the experiments of the tri-coexisting SEs system.

Figure 5 shows that the initial concentrations of E2 and EE2 in dual-combination are 13.3 and 14.9 µg·L^−1^, respectively, and the degradation ratios are 54.3% and 51.7% after 120 min. The initial concentrations of EE2 and E1 are 14.8 and 19.4 µg·L^−1^, respectively, and the degradation ratios of them are 53.0% and 49.2%, respectively. The initial concentrations of E2 and E1 in dual-combination pattern are 10.9 and 16.1 µg·L^−1^, respectively, and the degradation ratio of them are 55.6% and 50.7% after 120 min reaction.

**Table 4:** Parameters of degradation kinetic model of dual-mixed SEs.

Combinations	Constituent	Kinetic Equation	Reaction Rate Constant K/min^−1^	R^2^	Half-Life *t*_1/2_/min
E2+EE2	E2	ln(C_0_/*C*) = 0.0063*t* + 0.071	0.0063	0.967	99
	EE2	ln(C_0_/*C*) = 0.0059*t* + 0.058	0.0059	0.974	108
EE2+E1	EE2	ln(C_0_/*C*) = 0.0061*t* + 0.011	0.0061	0.990	112
	E1	ln(C_0_/*C*) = 0.0058*t* + 0.008	0.0058	0.992	118
E2+E1	E2	ln(C_0_/*C*) = 0.0070*t* + 0.035	0.0070	0.997	94
	E1	ln(C_0_/*C*) = 0.0061*t* + 0.038	0.0061	0.996	107

The results indicated that dual-combinations of E2, EE2, and E1 have better removal ratios than those in a tri-estrogens coexisting system, the degradation efficiency and reaction rate constant decrease with increasing numbers of constitute species and increasing total initial concentrations. The low concentration led to more effective degradation. In combined KMnO_4_/ultrasounds, the generation of the free hydroxyl radicals (•OH) is accompanied with the consumption. When the parameters of the KMnO_4_/ultrasound process were fixed, the yield of •OH was constant, and the collision probability between the molecules and •OH increases when a lower amount of target compounds joined the competition.

#### 3.3.4. Steroid Estrogens Removal by KMnO_4_/Ultrasound in Natural Water Background

**Figure 6:**
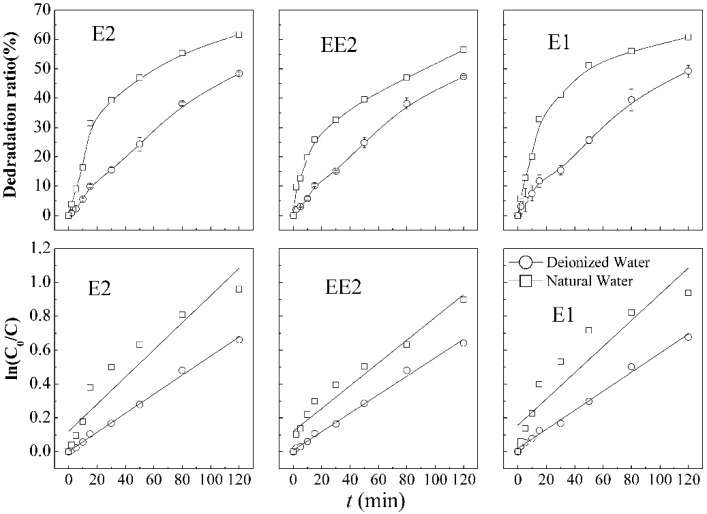
Steroid estrogens degradation curves and first order kinetic models in combination process under different aqueous background.

The competitive degradation for the tri-coexisting system occurring in raw surface water was studied, and the results were showed in Figure 6 and Table 6. The surface water was drawn from Shangtang River on campus, which had been pretreated by coagulation with PAC and sedimentation. The desired total concentration for target pollution is 50 µg·L^−1^. The characteristic of the pretreated natural water is shown in Table 5.

**Table 5:** The water quality parameters of the pretreated natural water.

Turbidity (NTU)	Color (CU)	Temperature (°C)	pH	TOC (mg·L^−1^)	UV_254_ (cm^−1^)
1.18	8	20	6.8	5.387	0.0396

The comparison of SEs removal efficiency in different backgrounds is shown in Figure 6. The degradation of E2 (49.2%), EE2 (48.4%), and E1 (47.4%) in pure water is lower than of E2 (61.8%), EE2 (56.5%), and E1 (60.8%) in natural water. The advantage was also reflected by the first order kinetic constants, with 0.00562 min^−1^ (E2), 0.00546 min^−1^ (EE2), 0.00563 min^−1^ (E1) of pure water and 0.00814 min^−1^ (E2), 0.00671 min^−1^ (EE2), and 0.00774 min^−1^ (E1) of natural water, respectively. The results demonstrate significant superior degradation in natural water background compared with those in pure water systems under the same process conditions.

**Table 6:** Parameters of kinetic model of degradation of steroid estrogens under different aqueous background.

Steroid Estrogens	Backgrounds	Kinetic Equation	Reaction Rate Constant K/min^−1^	R^2^	Half-Life *t*_1/2_/min
E2	Pure water	ln(C_0_/*C*) = 0.0056*t* + 0.003	0.0056	0.996	123
	Natural water	ln(C_0_/*C*) = 0.0080*t* + 0.122	0.0080	0.888	71
EE2	Pure water	ln(C_0_/*C*) = 0.0055*t* + 0.010	0.0055	0.994	124
	Natural water	ln(C_0_/*C*) = 0.0067*t* + 0.121	0.0067	0.944	100
E1	Pure water	ln(C_0_/*C*) = 0.0056*t* + 0.020	0.0056	0.993	120
	Natural water	ln(C_0_/*C*) = 0.0077*t* + 0.157	0.0077	0.867	70

In the natural water simulation system, the removal of estrogens increased significantly. The reason might be the presence of background components, including dissolved organic matter (DOM), cations, anions, organics *etc.*, enhancing the oxidation of estrogens by permanganate [44]. DOM was reported to generate photo-oxidants that can accelerate organic contaminants when irradiated at a solar wavelength of 254 nm [45]. The common bicarbonate ions were reported to be favorable in the elimination of micro-pollutants through carbonate radical producing, which can migrate towards the bulk of the solution and therefore induce the degradation, unlike the HO·radicals [46].

In a natural aquatic environment, the degradation rates of these three types of SEs increase prominently in 40 min compared with that of SEs in pure water, as illustrated in Figure 6, which also showed the removal rates decrease after that time point. Koyuncu *et al.* pointed out that when humic acid (10 mg·L^−1^) was added to the feed solution, the removal of the hormones increased to approximately 95% or greater with slightly higher values observed for the hormones alone [47]. These observations of enhanced removal of hormones in raw water can be explained by the influence of natural organic matter. Shao *et al.* reported the degradation of E1 by permanganate in different backgrounds and found that the removal efficiency of E1 in natural water is significantly better than that in an ultra-pure water system. Pétrier *et al.* [46] found that bicarbonate ion present in natural waters was favorable in the elimination of micro-pollutants through carbonate radical producing. Therefore, humic acid, reducing substances (SO_3_^2−^, NO^2−^ *et al*.), complexes (EDTA, citrate, oxalate), HCO_3_^−^, phosphate *et al.* obviously can promote the oxidation of steroid estrogens; appropriate common ions in natural water such as Mn^2+^, Fe^2+^ and Ca^2+^ can enhance the removal of E1, slightly; however, Al^3+^, Fe^3+^ and Mg^2+^ inhibit the degradation of estrogens during the permanganate oxidation process [28,44].

**Figure 7:**
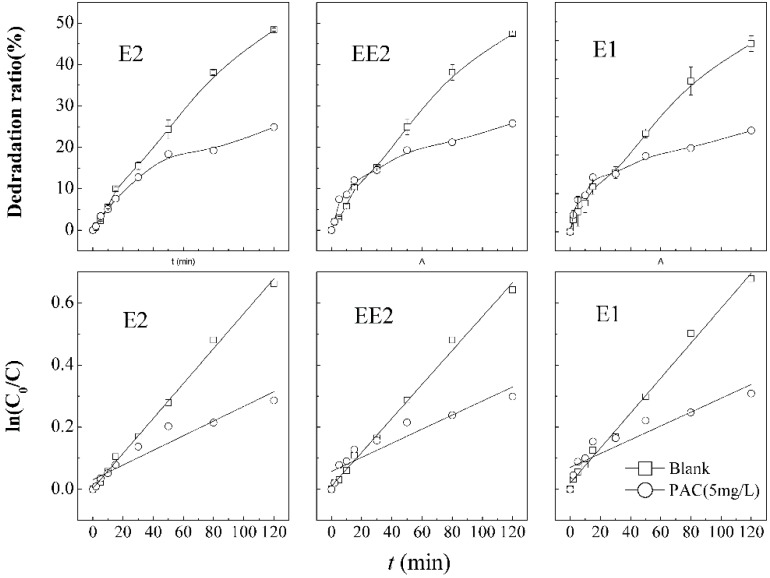
Degradation curves and first order kinetic models of SEs in the presence of Al^3+^.

In this experiment, coagulation with PAC (5 mg·L^−1^) was used to pre-treat the raw water, so it is unavoidable that Al^3+^ ions will be introduced into the system. Investigation of the impact of Al^3+^ ions on SEs degradation in the combined process was conducted and the results are shown in Figure 7. The degradation of E2, EE2, and E1 decreases sharply to 24.9%, 25.8%, and 26.5%, respectively, after adding the coagulant PAC. The results indicated that coagulation of PAC exerts an inhibitory effect on the degradation, because the excess of Al^3+^ ions produced in coagulation by the hydrolysis of PAC (Al_2_(OH)_n_Cl_6−n_, enhanced by ultrasound) had an adverse effect on the oxidation by the combined KMnO_4_/Ultrasound process, which is similar to the conclusion of Shao *et al.* [28].

The TOC values of the samples were collected at different times to measure the steroid estrogens’ mineralizing degree by combined KMnO_4_/ultrasound process.

**Figure 8:**
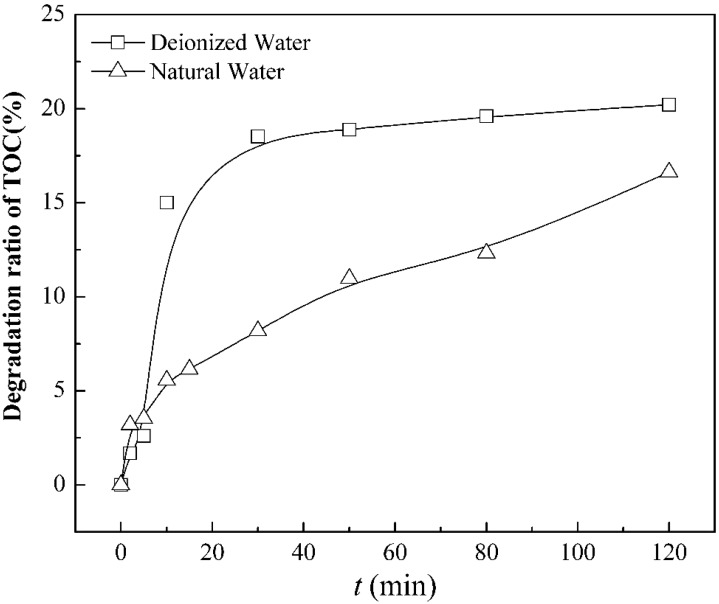
Mineralization of SEs indicated by TOC in KMnO_4_ /ultrasound process.

The results (presented in Figure 8 and Table 7) indicate that direct mineralizing degrees are unsatisfactory, with the TOC removal rate of 20.2% and 16.6% in natural water and pure water, respectively. In the combined simultaneous degradation system, the KMnO_4_/ultrasound process has effects on the removal of SEs, but still a large proportion of SEs remains. Instead, some intermediate products may be generated which still devote themselves to TOC. The oxidant capacity of •OH and KMnO_4_ is insufficient to completely decompose the steroid estrogens.

**Table 7:** Parameters of degradation kinetic model of SEs in the presence of Al^3+^.

Steroid Estrogens	Comparison of Condition	Kinetic Equation	Reaction Rate Constant K/min^−1^	R^2^	Half-Life*t*_1/2_/min
E2	Blank sample	ln(C_0_/*C*) = 0.0056*t* + 0.003	0.0056	0.9962	123
	Addition to PAC	ln(C_0_/*C*) = 0.0024*t* + 0.031	0.0024	0.9057	281
EE2	Blank sample	ln(C_0_/*C*) = 0.0055*t* + 0.010	0.0055	0.9939	125
	Addition to PAC	ln(C_0_/*C*) = 0.0023*t* + 0.057	0.0023	0.8526	281
E1	Blank sample	ln(C_0_/*C*) = 0.0056*t* + 0.020	0.0056	0.9931	120
	Addition to PAC	ln(C_0_/*C*) = 0.0022*t* + 0.070	0.0022	0.8337	280

## 4. Conclusions

Steroid estrogens (E2, EE2, and E1) in mixed aqueous systems can partly be removed by single KMnO_4_ and pure ultrasound irradiation, which are both less effective than oxidation by the combined KMnO_4_/ultrasound technique. In combined treatment processes, at a low initial concentration of μg/L, there was almost no significant competition of degradation between coexisting SEs.

The combined KMnO_4_/ultrasound process can remove the coexisting three steroid estrogens from an aqueous system more effectively as compared to the single processes. E2, EE2, and E1 in dual-combined simulated pollution show better removal efficiency than those in tri-estrogens coexisting systems. The degradation rates of SEs were significantly accelerated in a natural water background, which suggests that combined processes have prospective application in surface water treatment. However, it should be taken into consideration that Al^3+^ generated from coagulation has a negative effect on enhanced oxidation.

Further, with regard to coexisting SEs in water, it is not enough to focus on the degradation rate or removal efficiencies; more “sophisticated” toxicity tests, such as YES (yeas estrogen screen), and estrogenicity tests are needed in order to demonstrate the efficiency of a process.

## Abbreviations

Steroid estrogens (SEs); estrone (E1); 17β-estradiol (E2); 17α-ethinyl estradiol (EE2); solid phase extraction (SPE).
